# Consumer Preference and Willingness to Pay for Rice Attributes in China: Results of a Choice Experiment

**DOI:** 10.3390/foods13172774

**Published:** 2024-08-30

**Authors:** Pingping Fang, Zhou Zhou, Hua Wang, Lixia Zhang

**Affiliations:** 1 Institute of Agricultural Science and Technology Information, Shanghai Academy of Agricultural Sciences, Shanghai 201403, China; fangpingping@saas.sh.cn (P.F.); zhouzhou10629@163.com (Z.Z.); 2 Agro-Technology Extension Center of Fengxian District, Shanghai 201499, China

**Keywords:** rice attributes, consumption preference, willingness to pay (WTP), choice experiment method

## Abstract

Understanding urban consumers’ preferences for rice attributes is crucial for rice breeders, producers, and retailers to meet diverse and evolving market demands. Based on the sample data of 629 rice consumers in Shanghai, China, obtained through the choice experiment (CE) approach, this study uses the mixed logit (ML) model to analyze consumers’ preferences and willingness to pay (WTP) for food safety labels, brands, nutritional quality, and taste quality. Furthermore, the latent class (LC) model examines the heterogeneity in consumer group preferences. The research findings highlight that consumers prioritize taste quality as the most crucial attribute, followed by nutritional quality, food safety labels, and brand attributes. The premium rates for superior taste quality, organic certification labels, and green certification labels exceeded 100%. Interestingly, while combining organic certification with well-known international or domestic brands does not uniformly boost consumer preferences, incorporating green certification alongside well-known international or domestic brands significantly elevates those preference levels. Factors such as the external environment, consumption habits, and personal characteristics significantly influence individuals’ preferences for rice attributes. Based on these insights, the study puts forth policy recommendations for rice breeders, producers, and retailers.

## 1. Introduction

Food security is characterized by having physical, social, or economic access to an adequate, safe, and nutritious food supply that fulfills dietary requirements and preferences for a healthy and active life [1]. China is the world’s largest rice producer, consumer, and importer [2], with a significant proportion of its populace, roughly 65%, dependent on rice as a primary food source [3]. Following China’s reform and opening up, along with the rise in income levels of its population, there has been a surge in demand for high-quality staple foods among Chinese consumers. The significant price disparities observed across various types of rice in the Chinese market suggest that, even for staple foods like rice, with the improvement in income levels of Chinese residents, consumers are increasingly prioritizing quality over quantity in their food choices. However, governments sometimes prioritize investments in developing and making highly productive food crops more affordable, yet these may not always align with consumers’ preferences. Conversely, there may be inadequate support for so-called “orphan” crops or foods with specific quality attributes highly valued by consumers. Therefore, to ensure sustainable food security, policy initiatives must incorporate consumer preferences as a vital consideration.

**Figure 1:**
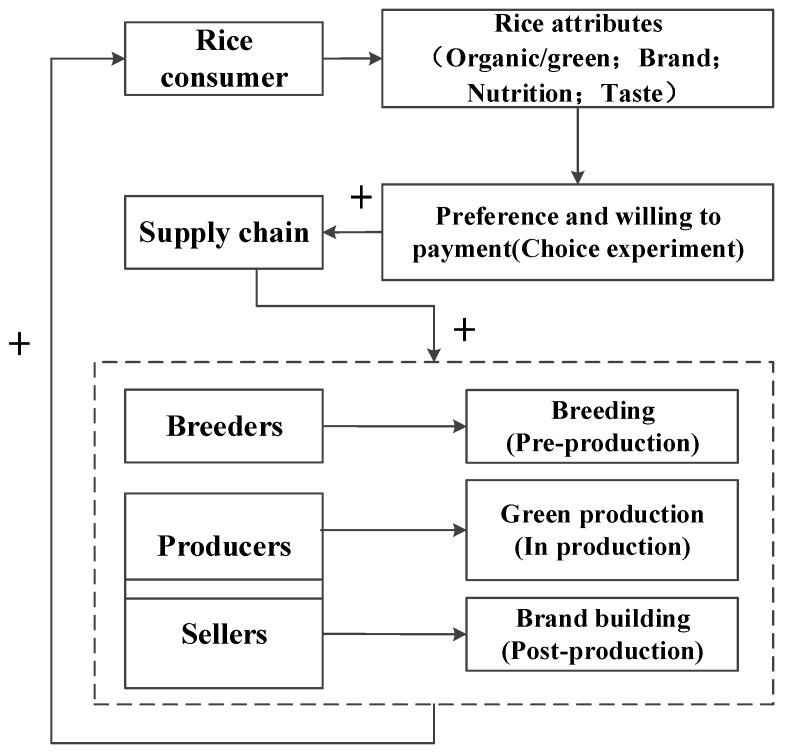
Conceptual framework.

In this study, our focus is on “preference matching” as a critical component of food security. We define it as access to diverse food options that allow consumers to align their food choices with their preferences, necessitating an analysis of consumer preferences and WTP. Specifically, we are concentrating on enhancing and customizing rice attributes to cater to the preferences of urban consumers through practices such as rice breeding, sustainable production methods, and strategic brand development. The conceptual framework outlining these concepts is encapsulated in Figure 1.

With a few exceptions [4,5,6], most existing studies investigating consumer WTP for various food attributes in the food sector focus on highly flexible food categories such as meat, fruit, and vegetables [7,8,9,10,11,12,13,14]. However, there is a noticeable gap in quantitative analyses regarding consumers’ WTP for staple food attributes, particularly concerning taste quality. This research trend overlooks the reality that, as living standards improve, consumers have begun to set higher expectations for the quality of staple foods. Specifically, an increasing number of consumers are paying more attention to attributes such as brand, variety, and taste quality, leading them to choose higher-quality staple foods.

This study employs the mixed logit (ML) model to analyze consumers’ preferences and WTP for food safety labels, brand recognition, nutritional quality, and taste quality based on data obtained through the choice experiment (CE) approach. Furthermore, the latent class (LC) model is employed to examine the heterogeneity in consumer group preferences. Through applying the CE method and subsequent analysis with the ML model, this research reveals how these factors influence consumer decisions and the relative importance assigned to each attribute. Furthermore, the use of the LC model highlights the diversity among consumer groups, demonstrating that not all consumers prioritize the same attributes when making food purchasing decisions. This finding emphasizes the necessity for tailored marketing strategies and product offerings that address the varied preferences of different consumer segments. The relevant conclusions will provide some references for the breeding direction of breeders, the variety selection and production methods of producers, and the marketing strategies of retailers.

## 2. Literature Review

Lancaster’s consumer theory posits that the utility derived from consuming a commodity stems from its attributes [15]. Building upon this theory, many studies have employed stated preference data to explore consumers’ inclinations and their valuation of different food attributes. These attributes are typically classified as either intrinsic or extrinsic [16]. Extrinsic attributes cover food labels, product types, appearance, and aroma, while intrinsic attributes are linked to taste quality and nutritional value.

### 2.1. Extrinsic Attributes

Extrinsic attributes, such as food labels, play a pivotal role by encompassing traceable labels, country-of-origin labels, safety labels, and branding. These labels contain information about intrinsic attributes and are considered suggestive attributes [9,17]. Regarding traceable labels, Vriezen et al. compiled a comprehensive literature review on consumers’ WTP for food traceability, encompassing 77 articles. Notably, the annual publication count of studies in this domain has generally increased during the review period. Three-quarters of these studies employed hypothetical methods to derive WTP values, with stated choice experiments being the most prevalent approach. In contrast, the remaining quarter utilized non-hypothetical methods, among which experimental auctions were the most common. These studies primarily focused on food categories such as meat (including pork and beef), dairy products, seafood, fruits, and vegetables [11]. Additionally, a meta-analysis of 72 peer-reviewed articles revealed a steady upward trend in consumers’ WTP for food traceability over time, culminating in a price premium of approximately 32% [18]. Regarding the country-of-origin labels, a study employed meta-regression analysis to synthesize heterogeneous outcomes from 204 WTP estimates for country-of-origin labels (COOL), extracted from 59 discrete choice experiments published between 2009 and 2020. The findings revealed a significant positive WTP for COOL on food products [19]. Regarding safety labels, a meta-analysis of 80 worldwide articles has revealed that consumers are willing to pay an average premium of 29.5% for sustainable products, primarily in categories such as meat, fruit, dairy, and vegetables [13].

In addition to food labels, research has explored other extrinsic attributes such as product type, appearance, and aroma. Studies suggest consumers often pay a premium for observable attributes [20]. Richardson et al. analyzed Haitian consumer data and employed hedonic pricing analysis to discover that Haitian consumers are not willing to pay more for reduced broken rice [5]. Bairagi et al. conducted rank-ordered logistic regression to identify significant factors influencing consumer demand for fragrant rice [21]. Moreover, Mottaleb et al. demonstrated through a multivariate unordered Probit model that affluent urban households and those with well-educated heads and spouses have a stronger preference for fine-grain rice compared to other households [22].

### 2.2. Intrinsic Attributes

Taste quality, which includes mouthfeel and flavor, emerges as a critical intrinsic attribute that affects consumer food demand. Existing research has mainly focused on consumers’ preferences and readiness to pay for the taste quality of fresh agricultural products, specifically fruits and vegetables. Several studies have emphasized that mouthfeel and flavor significantly influence consumers’ WTP for fresh agricultural produce [7,8].

Nutritional quality attributes represent another crucial intrinsic aspect. Traditionally, Chinese culture has embraced the idea of “medicine and food sharing the same origin”. With improved living standards, residents’ dietary habits have transitioned from simple to diverse, enhancing their understanding of nutritional balance and fostering a growing demand for nutritious, health-promoting foods [23]. Despite increasing studies on consumer WTP for nutritional quality attributes, they are still relatively limited. For instance, studies evaluating consumers’ preference for low-glycemic index rice have utilized self-reported measurement questionnaires and in-depth interviews [24].

### 2.3. Addressing Limitations and Contributing to the Existing Literature

While the existing literature provides a valuable foundation for further exploration, it does have some limitations. Firstly, there is a lack of comparative studies on how consumers prioritize taste quality over other food attributes, particularly concerning the taste quality of rice. Second, many investigations into consumer WTP in the food sector focus on highly flexible food categories such as meat and vegetables, with a shortage of quantitative analyses on the WTP for staple food attributes. Thirdly, there is only a little literature on rice attribute preference. Furthermore, these studies predominantly utilize discrete choice models, rank-ordered logistic regression, and the hedonic price function method (HPM) to assess rice attribute preferences. However, the first two methods are insufficient for quantitatively measuring WTP. The HPM, which relies on revealed preferences, is based on several stringent assumptions that may introduce biases in valuation due to bounded rationality and incomplete information [25].

This study makes two key contributions to the existing literature. First, it delves into consumers’ WTP for rice attributes, particularly focusing on taste quality while also evaluating other important food attributes. By addressing the research gap on taste quality evaluation, the study provides fresh insights that advance our understanding of consumer preferences and behaviors related to rice consumption. Second, the study enhances existing research on the WTP for staple foods through quantitative analysis utilizing data from the choice experiment (CE).

## 3. Materials and Methods

### 3.1. Choice Experiment

(1) Designing a Virtual Rice Product Based on the 5 kg Packaging Specification

After conducting investigations in supermarkets and rice-growing cooperatives, it was observed that small-packaged rice is predominantly available in 2.5 kg and 5 kg sizes. Therefore, this study opts for the more commonly found 5 kg packaging specification to create a virtual rice product.

(2) Selection of Experimental Attributes and Setting of Attribute Levels

We concentrated on food attributes, including food safety labels, brands, nutritional quality, and taste quality. Moreover, price is a crucial product attribute highlighted in the choice experiment questionnaire. It is a significant factor in buying decisions and often acts as an indicator of quality when information on food quality and risk assessment is limited [26]. Market surveys demonstrate the varying rice prices, with organic rice typically priced at around CNY 28 per kilogram and lower-quality rice at roughly CNY 6 per kilogram. Given that the average market price for 5 kg of rice hovers around CNY 50, this study sets a price range of CNY 25 to 150 per 5 kg, segmented into six price levels of CNY 25 each. Each choice scenario comprises two virtual rice products and a “Do not buy” option. Incorporating the “Do not buy” option can make the choice experiment closer to the natural situation. The absence of this option means that consumers will be forced to choose, and the probability of choosing each virtual product will be overestimated.

We conducted effect coding assignments for all attribute level variables to avoid confusion between the utility obtained by consumers selecting each attribute base level and the utility level represented by the “Do not buy” option [27]. The specific attribute levels and descriptions can be found in Table 1.

(3) Orthogonal experimental design

**Figure 2:**
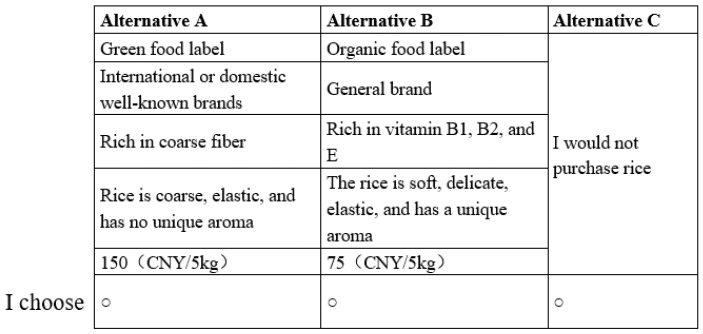
Example of a choice set used in the choice experiment questions.

Based on the attributes and levels outlined in Table 1, a full factorial design would theoretically yield 486 situations (3 × 3 × 3 × 3 × 6). However, it is unrealistic to require individuals to select combinations of attributes for so many scenarios. We adopted an orthogonal design approach to reduce the cognitive burden on consumers [28]. By leveraging SAS 9.4 software, a choice design consisting of 18 choice sets, each offering two alternatives, is crafted. Subsequently, the questionnaire is bifurcated into two versions, each comprising nine choice sets. This segmentation ensures that each interviewed consumer only needs to complete one version of the questionnaire, streamlining the data collection process. An illustrative choice task can be found in Figure 2.

### 3.2. Data Collection and Sampling

Shanghai, a megacity with a population exceeding 24 million, was chosen as the setting for this study due to its significant consumption of grains and its role as a major grain distribution hub in China. In 2021, the city consumed approximately 2.53 million tons of japonica rice, translating to 1.77 million tons of rice, while its local japonica rice production reached 740,900 tons, resulting in 518,600 tons after processing. As a result, about 71% of Shanghai’s demand for japonica rice depends on external procurement. Despite the potential for locally grown rice to meet a portion of the demand, field research indicates that Shanghai’s local rice faces sales challenges and struggles to capture a significant market share. The fierce competition from rice originating from other regions presents a substantial obstacle for Shanghai’s local rice in the market. Understanding the preferences of Shanghai consumers, their WTP for rice quality, and the overall demand can offer crucial insights for refining the rice breeding strategies of research institutions in the city, ultimately enhancing the profitability of rice cultivation.

In Shanghai, China, face-to-face interview surveys were carried out from July 2023 to September 2023, complemented by online surveys to ensure a comprehensive data collection approach. Prior to the main survey, the research team conducted an initial survey in Fengxian District and refined the questionnaire design based on the findings. Throughout the formal survey phase, the sample scope encompassed 15 Shanghai districts, excluding Huangpu District. Employing a random sampling technique, questionnaires were predominantly distributed in supermarkets and parks. Ultimately, the research team collected 706 questionnaires, of which 629 were considered valid, resulting in a questionnaire efficiency rate of 89.09%.

### 3.3. Individual Characteristic Variables and Descriptive Statistics

Table 2 illustrates the variable assignment and descriptive statistics of the personal characteristics of the surveyed consumers. Most participants were within the middle-aged bracket, aged between 30 and 49, constituting over 50% of the sample. Women accounted for 55.64% of respondents, slightly surpassing the male percentage. The educational background of most respondents spanned from college to master’s degree levels. Family sizes varied, with a prevalence of households comprising three, four, or five members, and fewer with two or fewer members. Most respondents reported monthly family incomes ranging from CNY 6000 to 30,000, making up 76.47% of the sample. On average, families purchased rice about 8.14 times annually.

Regarding hazard cognition of rice production, 36.41% of respondents assessed the environmental pollution arising from rice cultivation as “average,” indicating a moderate view. Furthermore, a combined 51.51% of respondents, with 29.89% holding the opinion that it is “very small” and 21.62% believing it to be “less”, conveyed a generally optimistic stance towards the environmental impact. Conversely, 8.59% found the pollution to be “large”, and 3.50% deemed it “very large”, highlighting a discernible contrast in perceptions of the severity of environmental pollution in rice production. Regarding safety awareness of organic rice, 38.47% of respondents expressed a “strong agreement”, and another 13.35% concurred with “agreement”, asserting that organic rice is safer than its conventional counterpart. Conversely, only 2.70% voiced “complete disagreement”, and 4.29% held “disagreement”, indicating a clear majority leaning towards the safety of organic rice.

### 3.4. Modeling Technique

This study utilizes the mixed logit (ML) model to estimate the consumer’s utility function, chosen for its ability to address the limitations present in the standard logit model. The ML model allows for the evaluation of consumers’ preferences and differences regarding various product attributes by eliminating assumptions of homogeneous preferences and limited substitutions between options among individuals [29]. The probability that individual n chooses alternative i in situation t is as follows:

(1) $$\bar{P_{nit}}=\int \frac{exp\left(V_{nit}\right)}{\sum_{j}exp\left(V_{njt}\right)}f\left(\beta |\theta \right)d\beta$$

Within the ML model, when assuming homogeneous preferences for a certain attribute, the attribute’s coefficient is designated as non-random; conversely, if there exists unobservable heterogeneity across individuals for a particular attribute, the coefficient is set as random. Building upon previous research [30,31], we posit that the coefficient for the price attribute remains non-random, while the coefficients for other attributes vary randomly among individuals.

Consumers’ WTP for a certain food attribute level refers to the highest premium that consumers are willing to pay to obtain that attribute level while keeping other food attribute levels unchanged and at least keeping their own utility unchanged. Suppose the consumer’s total utility is the linear sum of the utility represented by the various food attributes and prices. In that case, the formula for the consumer’s WTP is as follows:

(2) $${WTP}_{i}=-\frac{\beta_{i}}{\beta_{p}}$$

${WTP}_{i}$ is the willingness to pay for the i−h attribute level, $\beta_{i}$ is the mean estimation coefficient of that attribute level, $\beta_{p}$ is the estimation coefficient of price.

The importance of each attribute ($I_{i}$) is expressed by the difference between the maximum utility value and the minimum utility value in each level of the attribute, and the relative importance of each attribute ($Q_{i}$) is obtained by Equation (3) as follows:

(3) $$Q_{i}=I_{i}/\sum_{i}\left(I_{i}\right)$$

The greater the relative importance of an attribute, the more respondents value it.

Furthermore, this study employs a latent class model (LC) to categorize consumers with similar preferences and examine the heterogeneity in their WTP. The latent class model allows respondents to be grouped into distinct preference categories based on their individual and socioeconomic characteristics. Within each of these subcategories, consumer preferences exhibit homogeneity.

## 4. Results and Discussion

### 4.1. General Preferences for Various Rice Attributes

As displayed in Table 3, Chinese consumers exhibit a pronounced sensitivity towards pricing when making choices about rice. Indeed, the present observation concurs with the earlier findings of Xu et al., further validating the notion of Chinese consumers’ pronounced sensitivity towards rice pricing [32]. The coefficients for superior taste quality and medium taste quality exhibit significant positivity at the 1% level, with the coefficient for superior taste quality significantly outweighing that of medium taste quality. This indicates a clear consumer preference for taste quality. Alongside a preference for soft, glutinous, and elastic rice, consumers also like rice with a distinctive aroma. Regarding nutritional quality, consumers show a stronger preference for rice rich in vitamin B1, vitamin B2, and vitamin E over rice abundant in trace elements like potassium, magnesium, and calcium, with these preferences showing significantly positive coefficients at the 1% level; conversely, there is less enthusiasm for rice high in crude fiber content. Concerning food safety attributes, in comparison to no food safety label, the coefficients for organic certification labels and green certification labels are significantly positive at the 1% level, with the coefficient for green certification labels being higher. This indicates a stronger consumer preference for green certification labels, which could be attributed to lower trust or awareness of organic certification labels. Research shows that consumer trust in food labels determines WTP [33].

Based on Equation (3), we assess the relative importance of attributes that influence food selection at the point of sale and are essential to the success of new product development. Consumers rank taste quality as their top priority, followed by nutritional quality, food safety labels, and brand attributes. The significant focus on taste quality aligns with findings from numerous previous studies that have consistently identified taste quality as a key factor influencing consumer preferences and WTP for fresh agricultural products [7,34]. Notably, a study on Malaysian consumers by Ahmad Hanis et al. indicates that food safety is deemed the most critical attribute for rice, surpassing taste considerations [4]. Brand attributes are assigned lower importance, potentially due to their role as cue attributes influencing preferences for other independent attributes [9].

As depicted in Table 4, WTP for each attribute was computed using the data from Table 3, with the premium rates calculated against a base price of CNY 50 per 5 kg. The premium rates for superior taste quality, organic certification labels and green certification labels exceeded 100%. This academic finding not only reinforces the existing trend of consumer preferences gravitating towards high-quality and safe foods but also demonstrates the significant emphasis consumers place on taste and safety. Such insights are pivotal for discerning the evolving consumer landscape, predicting future consumption patterns, and informing adjustments to the agricultural industry’s structure. The substantial premium rates underscore consumers’ strong inclination to invest in superior quality rice that is not only delectable but also healthy, reflecting both the product’s inherent value and the market’s supply–demand equilibrium. This heightened WTP allows agricultural producers to garner premium prices and augment earnings by enhancing product quality and safety, driving agricultural industry transformation, and advancing sustainable development goals.

Moreover, the result highlights the viability of a market strategy centered on premium quality and pricing. Given consumers’ readiness to invest in top-notch rice, agricultural producers can elevate product value through refined cultivation techniques, robust quality control, and securing organic and green certifications, thereby commanding higher returns in the marketplace. This strategic approach not only caters to consumers’ quest for enhanced living standards but also propels the agricultural sector towards enhanced growth and prosperity.

### 4.2. Analysis of Synergistic Effect between Rice Brand and Safety Certification Labels

In this section, attributes interact to capture synergy between different attributes. This interactive effect can help us better understand individuals’ decision-making mechanisms in the selection process. Specifically, when two attributes interact, changes in one attribute will impact not only its own utility but also the utility of the other attributes. These interactive effects may result in substantial shifts in choice probabilities, providing a more precise description of an individual’s utility behavior during decision-making.

The data analysis results in Table 5 reveal a noteworthy negative correlation between the interaction of organic certification labels and well-known international or domestic brands. This finding indicates that consumers’ preference does not increase as expected when faced with rice carrying both organic certification and logos of well-known international or domestic brands; somewhat, their preference diminishes. In contrast, the interaction effect between green certification labels and well-known international or domestic brands demonstrates a positive correlation, indicating a synergistic relationship. Consequently, combining well-known brands with green certification leads to a significant enhancement in consumers’ preference levels. This phenomenon may stem from consumers’ skepticism or lack of trust in the credibility of well-known brands concerning organic certification. At the same time, they exhibit a higher level of trust in green certification.

In conclusion, the interaction effect analysis in this study unveils the intricate psychological mechanisms at play in consumers’ decision-making processes, offering a fresh lens through which to comprehend the interplay among brand trust, product certification, and consumer behavior. Subsequent research endeavors can delve deeper into the underlying factors driving these interaction effects, including the impact of consumer cognitive biases, information asymmetry, and other pertinent variables. Moreover, exploring methods to bolster consumer trust and embrace positive attributes like organic certification through impactful market communication and strategic brand development strategies will be vital for future investigations.

### 4.3. Analysis of Heterogeneity in Attribute Preferences and WTP for Rice

#### 4.3.1. Estimation Results Based on the ML Model

Results from the ML model showing estimations are displayed in Table 6. To begin, we analyze how the external environment affects consumers’ preference for organic certification labels. Interestingly, individuals living close to consumers who prefer organic rice demonstrate a stronger inclination toward organic certification labels. This phenomenon reflects the impact of social circles on personal choices, in line with the idea of peer effects, whereby others influence individuals [35,36], highlighting the interconnected nature of social dynamics. Additionally, consumers who view organic rice as safer than conventional rice exhibit a significantly higher preference for organic products. Previous research emphasizes that consumer attitudes towards organic foods, particularly related to health and environmental considerations, significantly influence consumer decision-making processes regarding organic food selections [37,38].

The discussion now shifts to analyzing the impact of consumption habits, framing them in terms of purchase frequency. Previous studies have shown that purchase frequency impacts the WTP for agricultural products [32,39]. Consumers who exhibit a higher frequency of rice purchases demonstrate a notably stronger preference for well-known local brands while displaying a diminished preference for well-known international or domestic brands. This phenomenon can likely be attributed to the heightened attention these frequent buyers place on cost-effectiveness, leading them to opt for affordable and dependable products. Leveraging their in-depth understanding of local market demands and consumer preferences, familiar local brands adeptly cater to the needs of such consumers, excelling in cost-effectiveness. Furthermore, local brands forge stronger connections with consumers through shared regional culture and taste preferences. Individuals making frequent rice purchases are more inclined to align their choice of rice brands with their lifestyle habits and taste inclinations, underscoring the innate advantage of well-known local brands in meeting these consumer preferences.

The focus now shifts to examining the influence of individual characteristics and family attributes on rice preferences. Older consumers tend to show a pronounced aversion to good-tasting rice. This preference may be linked to the decline in age-related taste functions and sensitivity [40,41]. Moreover, individuals with higher monthly household incomes exhibit a greater affinity for rice which is renowned for its superior taste profile. This preference is driven by the financial flexibility enjoyed by consumers in this group, enabling them to prioritize taste and quality over pricing considerations when selecting food products.

#### 4.3.2. Estimation Results Based on the LC Model

Before proceeding with the formal estimation, it is essential to determine the number of latent categories in the model. The Consistent Akaike Information Criterion (CAIC) and the Bayesian Information Criterion (BIC) are utilized for this determination. Lower CAIC and BIC values indicate a better-fitting model. As shown in Table 7, the CAIC and BIC values for the five, six, and seven categories are relatively similar. Excessive latent categories may diminish the sample size within each category, resulting in less distinct characteristics that are challenging to explain and articulate. Moreover, an excessive number of categories can complicate the analysis process. Thus, this study opts to classify the samples into five latent categories.

In addition, this study also added all the personal characteristic variables shown in Table 2 as covariates to determine the probability differences of consumers with different personal characteristics falling into each latent category. The estimation results of the LC model are shown in Table 8. Consumers are divided into “Strict requirement I”, “Food safety label preference”, “Price sensitive”, “Strict requirement II” and “Disregard food safety label”, accounting for 15.1%, 31.1%, 18.7%, 21.6%, and 13.5% of the total consumer sample, respectively. To facilitate the identification of coefficients within distinct classes, the membership coefficients pertaining to the final latent class undergo normalization to zero, thereby serving as a reference point for accurately assessing coefficients in the other classes.

Consumers are categorized as “Strict requirement I”, with distinct preferences for attributes related to food safety labels, nutritional quality, and taste. This preference is evident through their favoring of products bearing green certification labels, with claims such as “rich in vitamin B1, vitamin B2, vitamin E”, and a preference for rice with excellent or moderate taste profiles, with taste quality being the primary driver. Younger consumers with larger households are more likely to align with the “Strict requirement I” characteristics, potentially due to their heightened focus on nutrition, health, and taste. Moreover, the findings presented in Table 6 reaffirm that older consumers show a notably diminished preference for rice distinguished by superior taste quality.

Consumers classified under “Strict requirement II” clearly prefer all product attributes and exhibit heightened price sensitivity. An in-depth examination of the consumption patterns within this segment reveals a pronounced affinity for superior taste quality and products bearing green certification labels, reflecting a commitment to elevated quality of life and a deep-seated concern for health. A notable characteristic of this consumer group is the presence of individuals who are both high-income earners and relatively young in age. This correlation can be easily understood through the relationship between their high income and increased options [42,43,44]. With their financial resources and strong preferences, they can prioritize products that offer superior taste and safety features, emphasizing a pursuit that prioritizes not only material wealth but also a steadfast commitment to a health-conscious lifestyle.

Consumers categorized under “Food safety label preference” exhibit a diminished sensitivity to price, yet display a distinct inclination towards food safety certifications and well-known international or domestic brands. Their purchasing decisions prioritize overall product quality and brand reputation over price considerations. Food safety labels serve as a crucial reference point for them, underscoring their stringent stance towards food safety standards. Furthermore, they gravitate towards internationally renowned brands, symbolizing not just product quality and prestige but also their pursuit of a higher quality of life. Consumers who perceive organic rice as a safer alternative to conventional rice and reside close to those who prefer organic rice exhibit a notably heightened likelihood of belonging to this class.

Consumers classified as “Price-sensitive” exhibit heightened sensitivity to pricing and consistently seek the most cost-effective options available. However, their considerations extend beyond price alone. They emphasize food safety, meticulously scrutinizing and trusting products bearing food safety certifications. Additionally, they prefer well-known international or domestic brands, showcasing a pursuit of quality and recognition of brand prestige. Furthermore, they uphold stringent standards for food taste, favoring products with superior taste profiles. Intriguingly, individuals from larger households are more likely to fall within this consumer category, potentially due to the increased financial pressures associated with larger families, necessitating a balance between budget consciousness and a desire for quality and flavorful food.

Consumers categorized as “Disregard food safety label” demonstrate a heightened sensitivity to price fluctuations and consistently aim to optimize the price–performance ratio. Simultaneously, they strongly prefer the intrinsic quality of food, particularly favoring rice varieties rich in vitamins B1, B2, and E, and maintaining strict standards for taste quality, accepting only the best. Unlike other consumer segments, their attention towards food safety labels is relatively minimal, as their primary focus lies on the taste and nutritional content of the products. To them, a product’s taste and nutritional value serve as pivotal factors in their purchase decisions, with food safety labels viewed more as a formality than a determining factor in their choices.

## 5. Conclusions and Policy Implications

Based on a sample of 629 rice consumers in Shanghai, this study uses a choice experiment method to obtain data and analyze the preferences and WTP for food safety labels, brands, nutritional quality, and taste quality among consumers with different personal traits. In practical terms, the research findings can guide rice breeding, producers, and marketers regarding breeding direction, promotion, and marketing strategies. In academic terms, this study outlines the basic characteristics of rice consumption preferences among consumers in large Chinese cities, enriches the existing research on Chinese consumers’ preferences and WTP for staple food attributes, and provides a reference for future studies. The main conclusions of this paper are as follows.

The ML model confirms general preferences for various rice attributes. Taste quality is the most important factor, followed by nutritional quality attributes, food safety attributes, and brand attributes. The premium rates for superior taste quality, organic, and green certification labels exceeded 100%. Furthermore, when consumers face rice with both organic certification labels and well-known international or domestic brands, their preference does not increase as expected but weakens. In contrast, when well-known international or domestic brands are combined with green certification labels, consumers’ preference levels can be significantly improved. The research results indicate that consumers may doubt or distrust the authenticity of organic certification labels, while they have a high degree of trust in green certification.

The ML model also confirms the presence of preference heterogeneities among consumers. Factors such as the external environment, consumption habits, and personal characteristics significantly shape individuals’ preferences for rice attributes. Specifically, individuals residing near consumers who prefer organic rice show a heightened preference for organic certification labels. Furthermore, consumers who perceive organic rice as safer than conventional rice tend to favor organic products more strongly. Those who frequently purchase rice prefer well-known local brands and show less interest in well-known international or domestic brands. Additionally, older consumers often dislike rice with exceptional taste and quality; individuals with higher monthly household incomes tend to have a stronger affinity for rice known for its superior taste profile.

To examine the source of preference heterogeneities more carefully, the LC model is used to classify consumers based on the external environment, consumption habits, and personal characteristics. The model identifies five latent classes of consumers: “Strict requirement I”, “Food safety label preference”, “Price sensitive”, “Strict requirement II” and “Disregard food safety label”. Age, household scale, hazard cognition of rice production, safety cognition of organic rice, and community influence effectively determine class memberships.

Based on the above conclusions, we propose policy suggestions from three perspectives: rice breeders, producers, and sellers.

In the field of rice breeding, there is an urgent need to optimize and expand breeding strategies to meet consumers’ growing demand for superior taste quality. Data analysis indicates that consumers place the utmost importance on taste, making it the primary task for breeders to enhance the palatability of rice. Additionally, the analysis reveals that consumers prefer functional rice rich in vitamins B1, B2, and E. Therefore, breeding efforts should focus on nutrition and health-oriented approaches, aiming to genetically enhance the content of trace elements, vitamins, dietary fiber, and other health-promoting components in rice. This strategy will effectively cater to consumers’ pursuit of healthy eating habits.

In the rice production process, adopting an eco-friendly approach to rice production and management is essential while also considering economic benefits. Data indicate that consumers are willing to pay a premium for both organic and green certification labels. Therefore, promoting green planting methods that reduce chemical inputs is crucial. This shift not only enhances the safety and health attributes of the products but also aligns with the global trend toward sustainable development.

In the rice sales process, precision marketing strategies should be implemented. First, since data analysis shows that factors such as age, perception, income, and family size affect consumers’ WTP, market segmentation should be based on consumer personas to tailor rice product promotions to the needs of each segment. Second, since consumers’ WTP for green certification labels is higher than for organic certification labels, efforts should focus on strengthening the green product certification system while enhancing the criteria for organic certification. This practice will help improve consumer trust in organic products. Additionally, strategic cooperation among brands should be strengthened to leverage resource sharing and complementary advantages, creating a brand alliance with broad market influence that further enhances rice products’ competitiveness and brand value.

## Figures and Tables

**Table 1:** Text description and assignment for each level of selected experimental attribute indicators.

Attribute	Level	Describe	Variable Assignment
Food safety labels	3	Organic certification labels (Organic)	Organic = 1; Green = 0
		Green certification labels (Green)	Organic = 0; Green = 1
		No food safety label	Organic = −1; Green = −1
Brands	3	Well-known international or domestic brands (Brand1)	Brand1 = 1; Brand2 = 0
		Well-known local brands (Brand2)	Brand1 = 0; Brand2 = 1
		General brand	Brand1 = −1; Brand2 = −1
Nutritional quality (https://www.cn-healthcare.com/articlewm/20200101/content-1080903.html (accessed on 30 June 2023))	3	Rich in vitamin B1, B2, and E (Vitamin)	Vitamin = 1; Crude_fibre = 0
		Rich in coarse fiber (Crude_fibre)	Vitamin = 0; Crude_fibre = 1
		Rich in trace elements such as potassium, magnesium, and calcium	Vitamin = −1; Crude_fibre = −1
Taste quality	3	The rice is soft, delicate, elastic, and has a unique aroma (Taste1)	Taste1 = 1; Taste2 = 0
		The rice is soft, delicate, elastic, and has no unique aroma (Taste2)	Taste1 = 0; Taste2 = 1
		Rice is coarse, elastic, and has no unique aroma	Taste1 = −1; Taste2 = −1
Price	6	25; 50; 75; 100; 125; 150 (CNY per 5 kg)	
“Do not buy” option		The “Do not buy” option is selected: NONE = 1; The “Do not buy” option is not selected: NONE = 0	

**Table 2:** Descriptive statistics of consumption habits, consumption motivation, and personal characteristic variables.

Variables	Problem Setting	Description	Percentage (%)	Mean
Age	How old are you?	Under 19 years old (=1)	0.16	
		From 20 to 29 years old (=2)	22.42	
		From 30 to 39 years old (=3)	31.64	
		From 40 to 49 years old (=4)	23.69	
		From 50 to 59 years old (=5)	14.47	
		Over 60 years old (=6)	7.63	
Sex	What is your gender?	Female (=0)	55.64	
		Male (=1)	44.36	
Education	What is your education level?	Primary and below (=1)	1.43	
		Junior high school (=2)	5.41	
		High school/technical secondary school (=3)	8.90	
		Junior college (=4)	17.65	
		Bachelor’s (=5)	34.82	
		Master’s (=6)	23.53	
		Doctoral (=7)	8.27	
Household scale	How many families live together?	Two people or less (=1)	15.58	
		Three people (=2)	31.80	
		Four people (=3)	22.42	
		Five people and above (=4)	30.21	
Monthly household income	What is your monthly household income?	Less than CNY 3000 (=1)	3.34	
		From CNY 3000 to 6000 (=2)	7.47	
		From CNY 6000 to 10,000 (=3)	21.94	
		From CNY 10,000 to 15,000 (=5)	23.05	
		From CNY 15,000 to 20,000 (=7)	15.74	
		From CNY 20,000 to 30,000 (=10)	15.74	
		From CNY 30,000 to 50,000 (=14)	7.47	
		More than CNY 50,000 (=19)	5.25	
Purchase frequency	The number of times a family buys rice in a year	The specific number of times		8.14
Hazard cognition of rice production	How much pollution do you think rice production causes to the environment?	Small (=1)	29.89	
		Less (=2)	21.62	
		General (=3)	36.41	
		Greater (=4)	8.59	
		Big (=5)	3.50	
Safety cognition of organic rice	Do you think “organic rice” is safer than regular rice?	Complete disagreement (=1)	2.70	
		Disagreement (=2)	4.29	
		Uncertain (=3)	41.18	
		Agreement (=4)	38.47	
		Strong agreement (=5)	13.35	
Community influence	People you know around you eat organic rice	No (=0)	28.78	
		Yes (=1)	71.22	

**Table 3:** The difference in consumers’ preferences for various attributes of rice.

Attributes	Hierarchy Variable	Mean Estimation Coefficient	SD	Relative Importance (%)
	Price	−0.011 ***		
		(0.001)		
	“Do not buy” option	−2.646 ***	2.857 ***	
		(0.161)	(0.169)	
Food safety labels	Organic certification labels	0.567 ***	0.792 ***	14.00
		(0.048)	(0.053)	
	Green certification labels	0.715 ***	0.569 ***	
		(0.046)	(0.056)	
Brands	Well-known international or domestic brands	0.033	0.062	2.74
		(0.033)	(0.078)	
	Well-known local brands	0.062	−0.014	
		(0.040)	(0.052)	
Nutritional quality	Rich in vitamin B1, B2, and E	0.217 ***	0.035	33.40
		(0.040)	(0.056)	
	Rich in coarse fiber	−0.136 ***	0.055	
		(0.034)	(0.063)	
Taste quality	The rice is soft, delicate, elastic and has no unique aroma (Superior taste quality)	0.684 ***	0.664 ***	49.86
		(0.048)	(0.054)	
	The rice is soft, delicate, elastic and has no unique aroma (Medium taste quality)	0.157 ***	0.256 ***	
		(0.038)	(0.074)	

Notes: Standard errors in parentheses; *** *p* < 0.01.

**Table 4:** Consumers’ WTP for various attributes.

Attributes	Hierarchy Variable	WTP	z	95% Confidence Interval		Premium Rate (%)
Food safety labels	Organic certification labels	50.356 ***	9.540	40.009	60.702	100.71
	Green certification labels	63.468 ***	11.890	53.006	73.930	126.94
Brands	Well-known international or domestic brands	2.949	1.030	−2.684	8.582	5.90
	Well-known local brands	5.515	1.540	−1.493	12.523	11.03
Nutritional quality	Rich in vitamin B1, B2, and E	19.232 ***	5.290	12.102	26.362	38.46
	Rich in coarse fiber	−12.090 ***	−4.020	−17.989	−6.191	-
Taste quality	Superior taste quality	60.791 ***	11.060	50.020	71.562	121.58
	Medium taste quality	13.924 ***	4.050	7.177	20.670	27.85

Note: *** *p* < 0.01.

**Table 5:** Estimation of synergistic effects among various properties of rice.

Variables	Coefficients	Standard Errors
Price	−0.0103 ***	0.000829
Organic certification labels × Well-known international or domestic brands	−0.365 ***	0.0754
Organic certification labels × Well-known local brands	0.0817	0.0815
Green certification labels × Well-known international or domestic brands	0.199 ***	0.0687
Green certification labels × Well-known local brands	0.0881	0.0804
Attribute item	Yes	
Total	16,983	

Note: *** *p* < 0.01.

**Table 6:** Results of the ML model.

Variables	Coefficients	Standard Errors
Price	−0.0113 ***	0.000756
External environment		
Hazard cognition of rice production × Organic certification labels	0.0274	0.0400
Safety cognition of organic rice × Organic certification labels	0.170 ***	0.0516
Community influence × Organic certification labels	0.207 **	0.0984
Consumption habits		
Purchase frequency × Organic certification labels	0.00728	0.00827
Purchase frequency × Green certification labels	0.00529	0.00726
Purchase frequency × Well-known international or domestic brands	−0.0141 **	0.00587
Purchase frequency × Well-known local brands	0.0154 **	0.00710
Purchase frequency × Superior taste quality	0.00352	0.00767
Purchase frequency × Medium taste quality	−0.00216	0.00678
Personal characteristics		
Age × Organic certification labels	0.0266	0.0434
Age × Green certification labels	−0.0534	0.0393
Age × Well-known international or domestic brands	0.0292	0.0306
Age × Well-known local brands	−0.0254	0.0377
Age × Superior taste quality	−0.124 ***	0.0419
Age × Medium taste quality	−0.0102	0.0353
Sex × Organic certification labels	0.0518	0.0943
Sex × Green certification labels	−0.0686	0.0852
Sex × Well-known international or domestic brands	−0.0427	0.0659
Sex × Well-known local brands	0.0751	0.0804
Sex × Superior taste quality	−0.176 **	0.0881
Sex × Medium taste quality	−0.0515	0.0768
Education × Organic certification labels	0.0373	0.0398
Education × Green certification labels	−0.0192	0.0362
Education × Well-known international or domestic brands	−0.0166	0.0282
Education × Well-known local brands	−0.0144	0.0342
Education × Superior taste quality	−0.00425	0.0371
Education × Medium taste quality	−0.0212	0.0332
Household scale × Organic certification labels	−0.0303	0.0442
Household scale × Green certification labels	0.00718	0.0406
Household scale × Well-known international or domestic brands	−0.0199	0.0313
Household scale × Well-known local brands	0.0183	0.0382
Household scale × Superior taste quality	−0.0480	0.0414
Household scale × Medium taste quality	0.0216	0.0361
Monthly household income × Organic certification labels	0.00533	0.0110
Monthly household income × Green certification labels	0.00502	0.0101
Monthly household income × Well-known international or domestic brands	−0.000761	0.00790
Monthly household income × Well-known local brands	0.0136	0.00963
Monthly household income × Superior taste quality	0.0224 **	0.0104
Monthly household income × Medium taste quality	0.00858	0.00917
Attribute item	Yes	
Total	16,983	16,983

Note: *** *p* < 0.01, ** *p* < 0.05.

**Table 7:** Results of the adaptation index of the LC model.

Categorical Series	CAIC	BIC
2	8788.018	8694.691
3	8567.716	8425.504
4	8396.378	8205.281
5	8371.814	8131.831
6	8348.107	8059.238
7	8367.641	8029.887

**Table 8:** Results of the LC model.

	(1)	(2)	(3)	(4)	(5)
Variables	Strict Requirement I	Food Safety Label Preference	Price-Sensitive	Strict Requirement II	Disregard Food Safety Label
Utility function coefficients					
Price	0.00339 *	−0.00306	−0.0313 ***	−0.0114 ***	−0.0124 ***
	(0.00199)	(0.00244)	(0.00262)	(0.00230)	(0.00233)
None	−2.936 ***	−3.587 ***	−0.736 ***	−0.817 ***	−3.250 ***
	(0.621)	(0.444)	(0.161)	(0.202)	(0.325)
Organic certification labels	−0.0554	1.126 ***	0.648 ***	0.520 ***	0.0188
	(0.141)	(0.148)	(0.114)	(0.125)	(0.102)
Green certification labels	0.605 ***	0.806 ***	0.197 *	1.260 ***	−0.0710
	(0.169)	(0.102)	(0.107)	(0.143)	(0.0813)
Well-known international or domestic brands	−0.0749	0.218 **	0.215 **	−0.158	−0.0296
	(0.111)	(0.0913)	(0.105)	(0.114)	(0.0960)
Well-known local brands	0.0752	0.0650	−0.122	0.258 **	0.0496
	(0.105)	(0.115)	(0.114)	(0.108)	(0.0900)
Rich in vitamin B1, B2, and E	0.283 **	0.0225	0.135	0.303 ***	0.174 **
	(0.133)	(0.0972)	(0.107)	(0.118)	(0.0816)
Rich in coarse fiber	−0.297 ***	0.0711	−0.179	−0.194 **	−0.0256
	(0.108)	(0.0729)	(0.113)	(0.0966)	(0.0823)
Superior taste quality	1.003 ***	0.126	0.491 ***	1.339 ***	0.202 **
	(0.199)	(0.101)	(0.103)	(0.143)	(0.0898)
Medium taste quality	0.364 ***	−0.0849	−0.0155	0.641 ***	−0.106
	(0.0988)	(0.131)	(0.114)	(0.122)	(0.0895)
Class membership Coefficients					
Hazard cognition of rice production	0.421 **	0.0629	0.161	0.230	
	(0.189)	(0.161)	(0.162)	(0.164)	
Safety cognition of organic rice	0.296	0.323 *	−0.0882	0.149	
	(0.266)	(0.195)	(0.195)	(0.202)	
Community influence	0.742	0.892 **	0.191	−0.0123	
	(0.465)	(0.376)	(0.354)	(0.369)	
Purchase frequency	−0.00191	0.00406	−0.0142	0.0234	
	(0.0362)	(0.0294)	(0.0330)	(0.0313)	
Age	−0.481 **	−0.132	−0.160	−0.293 *	
	(0.196)	(0.156)	(0.156)	(0.160)	
Sex	−0.624	0.167	0.246	−0.200	
	(0.423)	(0.342)	(0.342)	(0.352)	
Education	−0.147	0.0979	−0.0965	0.167	
	(0.190)	(0.147)	(0.149)	(0.160)	
Household scale	0.584 ***	0.320 **	0.352 **	0.0111	
	(0.208)	(0.161)	(0.165)	(0.172)	
Monthly household income	−0.00253	−0.00601	0.0152	0.0893 **	
	(0.0530)	(0.0421)	(0.0430)	(0.0415)	
Constant	−1.409	−1.978	0.158	−1.132	
	(1.760)	(1.497)	(1.433)	(1.513)	
Observations	16,983	16,983	16,983	16,983	16,983
Number of groups	5661	5661	5661	5661	5661
Latent class share	15.1%	31.1%	18.7%	21.6%	13.5%

Notes: Standard errors in parentheses; *** *p* < 0.01, ** *p* < 0.05, * *p* < 0.1.

## Data Availability

The original contributions presented in the study are included in the article; further inquiries can be directed to the corresponding author.
